# Case report: Identification of a Chinese patient with *RAG1* mutations initially presenting as autoimmune hemolytic anemia

**DOI:** 10.3389/fimmu.2024.1498066

**Published:** 2024-12-10

**Authors:** Xin Chen, Chunxue Jiang, Wenliang Song, Tingting Sun, Jingli Yan, Wei Xu, Kai You

**Affiliations:** Department of Pediatrics, Shengjing Hospital of China Medical University, Shenyang, China

**Keywords:** RAG1, autoimmune hemolytic anemia, V(D)J Recombination, case report, literature review

## Abstract

Mutations in the recombination-activating gene 1, a pivotal component essential for V(D)J recombination and the formation of T- and B-cell receptors, can result in autoimmune hemolytic anemia, a rare hematological condition characterized by the autoantibody-mediated destruction of red blood cells. Herein, we report the case of a 1-year-and-4-month-old girl who presented with progressively aggravated anemia, fever, and cough. Autoimmune hemolytic anemia was confirmed by bone marrow aspiration and Coombs test. During treatment, the patient experienced two episodes of severe pneumonia and respiratory failure. Next-generation metagenomic sequencing of sputum samples confirmed the presence of cytomegalovirus and *Pneumocystis jirovecii* infections. Additionally, lymphocyte subset analysis revealed a T-B+ immunodeficiency. Whole exome and Sanger sequencing revealed a pathogenic recombinase-activating gene 1 mutation (c.2095C>T, p.Arg699Trp) and a likely pathogenic variant (c.2690G>A, p.Arg897Gln), resulting in a missense mutation in the amino acid sequence of the coding protein. Consequently, the patient was diagnosed with a recombination-activating gene 1 mutation and autoimmune hemolytic anemia as the initial presentation. This study reports a case of a recombination-activating gene 1 mutation in China and documents a combination of mutation sites and associated clinical phenotypes that were previously unreported. In this study, we outline the diverse clinical phenotypes observed in cases of recombination-activating gene 1 mutations presenting with autoimmune hemolytic anemia, aiming to facilitate timely diagnosis and appropriate treatment.

## 1 Introduction

The recombination-activating gene 1 (*RAG1*) is a pivotal element in V (D) J (V:Variable;D:Diversity;J:Joining) recombination (1). Mutations in this gene disrupt the normal formation of T- and B-cell antigen receptors, adversely affecting the functionality of the immune system. The incidence of *RAG1/2* gene mutations is estimated to be approximately 1.8 – 3 cases per 100,000 newborns (2–4). Mutations in *RAG1* can lead to immune dysregulation (5, 6), which can manifest as severe opportunistic infections and pose a risk of autoimmune manifestations (7, 8). Among them, autoimmune hemolytic anemia (AIHA), has been reported as a rare clinical manifestation of immune dysregulation, albeit in a limited number of cases (9). AIHA is an uncommon hematological disorder characterized by the binding of autoantibodies to the surface membrane of red blood cells, resulting in premature destruction (9). Its clinical manifestations range from mild to life-threatening anemia. Therefore, early comprehensive diagnosis, timely hemolysis control measures, and subsequent allogeneic stem cell transplantation are essential for AIHA caused by *RAG1* mutation.

Here, we present the case of a *RAG1* mutation in a Chinese patient, which initially manifested as AIHA, with an associated mutation site combination and its clinical phenotype, which have not been previously reported. Additionally, through a literature review, we have summarized the key clinical information and genotypes of all reported cases of *RAG1* mutations causing AIHA, to enhance understanding of early identification, disease progression, and timely initiation of immune reconstitution therapy, as well as to support genetic counseling.

## 2 Case description

**Figure 1:**
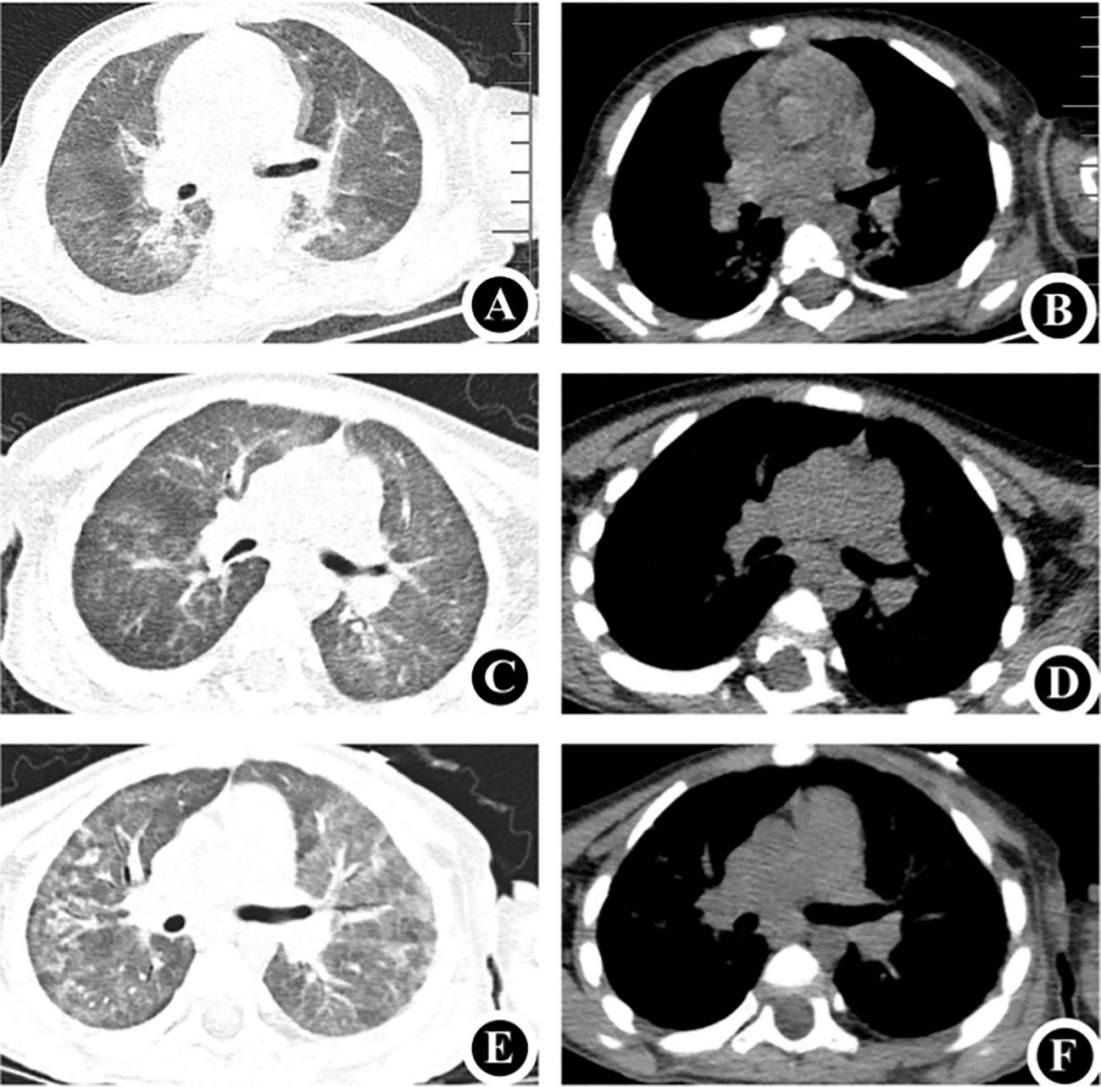
Chest CT **(A, B)** May 5, 2024: Demonstrates uneven bilateral lung transparency with multiple ground-glass opacity (GGO) patchy shadows, consistent with the manifestations of *CMV*. **(C, D)** June 5, 2024: Compared to the previous scans, a noticeable decrease was observed in the extent of GGO patchy shadows in both lungs. **(E, F)** August 5, 2024: Reveals a decrease in bilateral lung field transparency and an increase in multiple GGO patchy shadows, consistent with the typical manifestations of *Pneumocystis jirovecii* pneumonia. CT, computed tomography.

A 1-year-and-4-month-old female, born full-term via cesarean section and weighing 3860 g at birth, is the first child of unrelated, healthy parents. She had previously been in good health; however, on May 5, 2024, she was admitted to the hospital with a persistent fever and progressively worsening anemia. Upon admission, a decline in hemoglobin levels was observed, accompanied by an elevation in reticulocyte count and percentage. A positive Coombs test and bone marrow cytology findings confirmed the diagnosis of AIHA (**Supplementary Figure 1**). After treatment with hormones, intravenous immunoglobulins (IVIG), intermittent low-volume blood transfusions, and plasmapheresis, the patient’s anemia and hemolytic symptoms gradually improved. Nonetheless, transient decreases in white blood cell and granulocyte counts were observed. During treatment, the patient developed a cough and progressively aggravated dyspnea. Targeted next-generation sequencing (tNGS) of sputum samples confirmed the presence of cytomegalovirus (*CMV*), while chest computed tomography (CT) scans revealed associated abnormalities or lesions (**Figures 1A, B**).

The patient was administered ventilator-assisted ventilation and ganciclovir as *CMV* antiviral therapy. Mechanical ventilation was discontinued on day six of admission. By the 18th day of hospitalization, the patient’s symptoms had gradually resolved, allowing for discharge. After discharge, the patient was prescribed intermittent low-dose glucocorticoid therapy. One month later, follow-up chest CT at the outpatient clinic indicated significant improvement in the bilateral lung inflammation (**Figures 1C, D**). Routine blood re-examination revealed no signs of hemolysis.

However, two months after discharge, the patient was admitted to hospital on July 31, 2024, due to dyspnea accompanied by retraction and cyanosis of the upper extremities. The patient was conscious but in poor general condition, exhibiting tachypnea, nasal flaring, and three positive concave signs. Immediately upon admission, the patient was intubated and provided ventilator-assisted ventilation. The tNGS of sputum revealed *Pneumocystis jirovecii* infection, and the chest CT showed findings shown in **Figures 1E, F**. Immunological assessment showed an immunoglobulin G level of 6.17 g/L (7.51–15.6), immunoglobulin A level of 0.2 g/L (0.82–4.53), and immunoglobulin M level of 0.51 g/L (0.46–3.04). Lymphocyte subset analysis revealed that T, natural killer, and B lymphocytes accounted for 17.19% (55–84), 35.18% (7–36), and 44.18% (5–10) of lymphocytes, respectively. Helper and suppressor T lymphocytes accounted for 0.45% (31–60) and 5.19% (13–41) of the total lymphocytes, respectively (**Table 1**). These results confirmed the possibility of T-B+ immunodeficiency. Therefore, the patient was treated with trimethoprim-sulfamethoxazole, and the dyspnea gradually improved. In both hospitalizations of the patient, the patient’s inflammatory markers were continuously monitored, and the specific trends and values are presented in **Supplementary Figure 2**.

**Table 1:** Immunological data upon admission and before discharge.

Variable	Admission in May, 2024	Admission in July, 2024	Reference range
Serum immunoglobulins			
Immunoglobulin G (g/L)	10.23	6.17	7.51–15.6
Immunoglobulin M (g/L)	3.72	0.51	0.46–3.04
Immunoglobulin A (g/L)	0.4	0.2	0.82–4.53
Lymphocyte subsets			
CD3+ (%)	5.80	17.19	55–84
CD3+ (cells/μL)	84	73	690–2540
CD3+CD8+ (%)	5.78	5.19	13–41
CD3+CD8+ (cells/μL)	38	22	190–1140
CD3+CD4+ (%)	1.17	0.45	31–60
CD3+CD4+ (cells/μL)	8	2	410–1590
CD16+CD56+ (%)	35.34	35.18	7–36
CD16+CD56+ (cells/μL)	233	150	90–590
CD19+ (%)	48.93	44.18	5–10
CD19+ (cells/μL)	323	189	90–660
CD4+/CD8+	0.20	0.09	0.71–2.78

**Figure 2:**
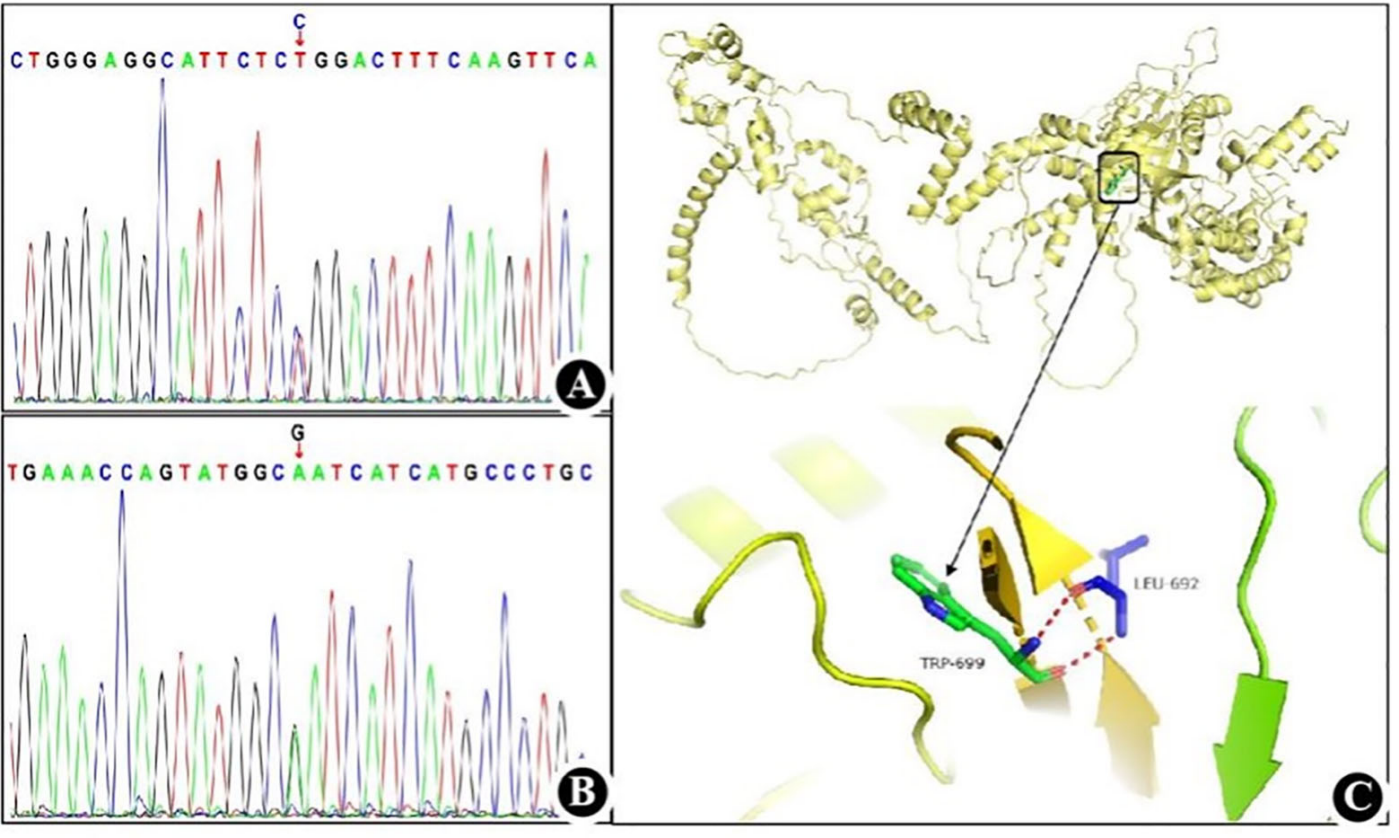
*RAG1* mutation detected in the present case **(A)** Sanger chromatogram of the pathogenic variant. **(B)** Sanger chromatogram of the likely pathogenic variant. **(C)** Three-dimensional structure diagram of protein after *RAG1* pathogenic mutation.

Given the patient’s history of autosomal hypopomorphic immunodeficiency associated with AIHA, and past infections with *CMV* and *Pneumocystis jirovecii*, we performed genetic testing of the patient and her parents to identify the molecular and genetic risk of immunodeficiency (**Figure 2**). Whole-exome and Sanger sequencing identified a substitution mutation (c.2095C>T) in exon 2 of *RAG1* (chr11:36596949), leading to a missense mutation at the amino acid level in the encoded *RAG1* protein (NM_000448.3). This mutation results in substituting the 699th amino acid, arginine (Arg), with tryptophan, denoted as p.Arg699Trp, which is classified as a pathogenic variant. This mutation was classified as a pathogenic variant, is rare with a gnomAD frequency of 0.0000080, and reported as deleterious by the Combined Annotation Dependent Depletion (CADD) score of 22.8. Experimental data indicated that the patient carried the mutation in a heterozygous state, specifically inheriting it from her mother, while the father had a wild-type at this locus. Furthermore, we identified a likely pathogenic variant [NM_000448.3(*RAG1*):c.2690(exon2)G>A; p.(Arg897Gln)], in which a substitution mutation occurred at nucleotide position 2690 within the coding sequence of the *RAG1* gene. This led to a missense mutation, causing the 897th amino acid to be replaced with glutamine (Gln). **Figure 2** presents the two components: 1) the Sanger chromatogram depicts both pathogenic and likely pathogenic variants; 2) a three-dimensional structural diagram of the protein post-mutation showcase the pathogenic and likely pathogenic variants of *RAG1*, which were generated using PyMOL 2.1.0 (PyMol Molecular Graphics System, Schrödinger, LLC). This study reports a case of a recombination-activating gene 1 mutation in China and documents a combination of mutation sites and associated clinical phenotypes that have not been reported previously.

The patient had received Bacillus Calmette-Guérin (BCG) vaccine at birth. During the observation period of this study, the patient did not exhibit symptoms of BCG disease nor signs of tuberculosis infection. Up to the final follow-up, the patient continued to receive IVIG replacement therapy and antibiotic prophylaxis.

## 3 Systematic review

A comprehensive search was undertaken across the Web of Science, ClinVar, Embase, and Medline databases, using the search terms “*RAG* “ and “hemolytic”. In June 2024, this study compiled and published findings from 13 reports encompassing 41 children diagnosed with AIHA resulting from mutations in the *RAG 1* gene (**Table 2**). Among the 41 children (included in current and prior studies), 22 (54%) were male. With the exception of one patient whose age remained unascertained, the median age at diagnosis was 3.75 years (range, 1 month – 17 years).

**Table 2:** Clinical and serological characteristics of patients with distinct phenotypes of RAG deficiency.

CODE	Phenotype	Mutation		Sex	Age at clinical diagnosis	Clinical autoimmunity	Location of granulomas	Other clinical manifestations/infection	On IvIg	Other immune modulation	HCT	Current status Age (cause of death)	Clinical autoantibodies (+)	Reference
L/AS-1	Leaky/atypical SCID	*RAG1*	a.2095(exon2)C>T b.2690(exon2)G>A	F	1y 4m	AIHA; AN	_	Severe pneumonia; *CMV* infection; *pneumocystis jirovecii* disease	yes	high-dose steroid and plasmapheresis	n.a.	Alive; 1y 7m	Coombs	Present case
L/AS-2	Leaky/atypical SCID	*RAG1*	a. R669W b. M435V	M	5 y	AIHA; ITP; AN	_	pneumonia; sinus infection; oral candidiasis; vitiligo; psoriasis; Guillain-Barré sy	yes	_	yes; 6y 6m	Alive; 14y 11m	Coombs; anti-platelet ab; anti-neutrophil ab; anti-TPO ab IBD serology+	(11)
L/AS-3	Leaky/atypical SCID	*RAG1*	a./b. p.R108*	F	3 m	AIHA	_	*CMV*(+); dermatitis	yes; pre-HCT	_	yes; 6.7m	Alive; 2y 7m	Coombs	(11)
L/AS-4	leaky/atypical SCID	*RAG1*	a./b. K86fs*33	M	11 m	AIHA	_	pneumonia; chronic *CMV* infection; Miller Fisher Syndrome (polyradiculoneuritis with cranial nerve involvement)	yes; pre-HCT	high-dose steroid; rituximab; plasmapheresis	yes;1y 6m	Alive; 3y 4m	Coombs; anti-platelet ab	(11)
L/AS-5	Leaky/atypical SCID	*RAG1*	a. H65R b. A857V	F	2 y	AIHA	_	adenovirus; *CMV*(+)	yes	_	n.a.	Alive; 3y 1m	Coombs	(11)
L/AS-6	Leaky/atypical SCID	*RAG1*	a./b. R841W	M	9m	AIHA	_	_	_	_	yes; 10m	Died; 1y 9m	Coombs	(5)
L/AS-7	Leaky/atypical SCID	*RAG1*	a./b. R474H	F	11y	AIHA; ITP	_	amyloidosis	_	_	n.a.	Alive; 5y	Coombs	(5)
L/AS-8	Leaky/atypical SCID	*RAG1*	a.1420C(exon2)>T het b.2949(exon2)delA het	F	3y 6m	AIHA	_	recurrent viral and bacterial infections; and nephrotic syndrome	yes; pre-HCT	busulfan; fludarabine and anti-thymocyte globulin	yes; 8 y	Alive	Coombs; Lupus anticoagulant + ANCA; +anti-MPO; Thyroglobulin and thyroid peroxidase Ab; Anti-IFNα Ab	(12)
L/AS-9	Leaky/atypical SCID	*RAG1*	a.1420C(exon2)>Thet b.2949(exon2)delA het	F	2y	AIHA; AN	_	eczematous rash	yes; pre-HCT	busulfan; fludarabine and anti-thymocyte globulin	HSCT; 2y 5m	Alive	Coombs (IgG and complement); Anti-neutrophil Ab; Anti-IFNα Ab	(12)
L/AS-10	Leaky/atypical SCID	*RAG1*	a. W522C b. M435V/M1006 V	M	2y	AIHA; ITP	skin	_	yes; pre-HCT	rituximab	yes;3y	Alive	Coombs; platelet	(5)
L/AS-11	Leaky/atypical SCID	*RAG1*	a. R396C b. M435V	M	1y 6m	AIHA	_	vasculitis	yes; pre-HCT	steroids; IVIG; cyclophosphamide; alemtuzumab; and/or rituximab	yes;1y 9m	Died; 2y 8m	Coombs; IFN-α	(5)
L/AS-12	Leaky/atypical SCID	*RAG1*	a. R561H b. R778Q	M	17y	AIHA	_	_	_	_	_	Alive; 17y	Coombs	(5)
L/AS-13	Leaky/atypical SCID	*RAG1*	a. N855S b. K992E	M	2y 9m	AIHA	_	hepatitis	_	_	yes; 3y	Alive; 3y	Coombs	(5)
L/AS-14	Leaky/atypical SCID	*RAG1*	a. R142* b. T477S	F	2y 10m	AIHA; ITP; AN	_	_	_	_	yes; 3y 3m	Died; 3y 4m	Coombs; platelet	(5)
L/AS-15	Leaky/atypical SCID	*RAG1*	a. R15L b. H735Q	F	n.a.	AIHA; ITP	_	−	_	_	yes; 1y 4m	Alive; 3y 9m	Coombs; thyroid (TPO)	(5)
L/AS-16	Leaky/atypical SCID	*RAG1*	n.a.	M	14y	AN; ITP; AIHA	_	vitiligo; IBD	_	_	yes; 14y	Died; 14y	Coombs	(5)
CID-1	CID-G/AI	*RAG1*	a. R474C b. K983Nfs*9	F	2y	AIHA; AN	_	otitis media; RSV bronchiolitis; severe varicella with periorbital cellulits/keratitis	yes; pre-HCT	_	yes; 2 and 4 y	Alive; 3m	Coombs;anti-neutrophil antibody+	(11, 12, 13, 22)
CID-2	CID-G/AI	*RAG1*	a./b.S117fs	M	8y	AIHA	skin	pneumonias; liver abscess; Mycobacterial osteomyelitis; herpes-zoster; diarrhea from rotavirus viral encephalitis after yellow fever vaccine; varicella	yes; pre-HCT	_	yes;8 y	Alive; 8y	Coombs	(10)
CID-3	CID-G/AI	*RAG1*	a. K86fs*33 b. H65R	F	3y	ITP; AIHA	lung	recurrent sinopulmonary infections (*Penicillium*; *Corynebacterium propinquum*; and *Pseudomonas aeruginosa*); viral infections (shingles); and vitiligo)	yes; pre-HCT	corticosteroids and infliximab	yes; 18y	Alive; 20y	Coombs; thyroid (TPO)	(13)
CID-4	CID-G/AI	*RAG1*	a./b. S480G	M	6y	AIHA; AN	_	Enteropathy;Glomerulonephritis; atherosclerosis;generalized urticaria	yes; pre-HCT	rituximab	yes; 9y 6m	Died; 10y	Coombs; neutrophil	(14)
CID-5	CID-G/AI	*RAG1*	a./b.[His65Arg]	M	7y	AIHA; ITP	skin	Severe varicella infection; generalized scabies; recurrent bronchitis	yes; pre-HCT	rituximab	yes; 7y 6m	Alive	Coombs	(14)
CID-6	CID-G/AI	*RAG1*	a./b.2095C>T	M	6y	AIHA	skin, lung, liver, bone, pancreas, testes	otitis media; pallor and splenomegaly	yes; pre-HCT	high-dose steroid; rituximab; plasmapheresis	n.a.	Died	Coombs; ANA; dsDNA	(15)
CID-7	CID-G/AI	*RAG1*	a. R474C b. R975W	F	9y	AIHA; ITP	skin	_	yes; pre-HCT	rituximab	yes; 20y	Died; 21y	Coombs; platelet	(5)
CID-8	CID-G/AI	*RAG1*	a. W522C b. H994R	M	3y	AIHA	_	vasculitis	yes; pre-HCT	steroids; cyclophosphamide; alemtuzumab; and/or rituximab	yes;5y	Died; 6y	Coombs	(5)
CID-9	CID-G/AI	*RAG1*	a./b. R764C	F	16y	AIHA; ITP	skin, bone	splenomegaly; hypergamma; globulinemia; pancytopenia	_	_	n.a.	Alive	Coombs	(16)
CID-10	CID-G/AI	*RAG1*	a. R841Q b. F974L	F	6m	AIHA; ITP; AN	_	vasculitis; myopathy; central demyelinating neuropathy	yes	glucocorticoids; intravenous immunoglobulin; enoxaparin; and rituximab	died	Died; 2y	Coombs; platelet	(17)
CID-11	CID-G/AI	*RAG1*	a./b. H65R	F	15y 7m	AIHA; AN	_	alopecia areata; thyroiditis	yes; pre-HCT	_	yes; 17y	Alive; 18y	Coombs; IFN-α; thyroid (TPO/TG)	(5)
CID-5	CID-G/AI	*RAG1*	a./b. R507G	M	5y	AIHA; AN	_	hepato-splenomegaly	yes; pre-HCT	high-dose steroid; rituximab; plasmapheresis	yes; 5y 3m	Alive; 8y	Coombs; neutrophil	(18)
CID-13	CID-G/AI	*RAG1*	a./b. K86VfsX33	M	4y	AIHA; ITP; AN	_	_	yes; pre-HCT	_	yes; 4y	Alive; 7y 8m	Coombs	(5)
CID-14	CID-G/AI	*RAG1*	a./b. N855S	M	11y	AIHA	_	enteropathy	none	_	n.a.	Died; 11y	Coombs; enterocyte/goblet cell	(5)
CID-15	CID-G/AI	*RAG1*	a./b. G816R	M	7y 6m	AIHA	_	sclerosing cholangitis	_	_	n.a.	Alive; 9y 6m	Coombs	(5)
CID-16	CID-G/AI	*RAG1*	a.1187G>A (Arg396His) b.1566G>T (Trp522Cys)	F	5y	AIHA	skin	herpes simplex virus (HSV) gingivostomatitis; bacterial pneumonia; and cutaneous abscess	yes; pre-HCT	high-dose steroid; rituximab; plasmapheresis	yes	Alive	Coombs	(19)
CID-17	CID-G/AI	*RAG1*	a.M435V (1303AG) b.R699W (2095C¡T)	M	10m	AIHA; ITP; AN	_	vitiligo; psoriasis;tetanus; diphtheria and Guillain-Barré syndrome	yes; pre-HCT	high-dose steroids; infliximab; and cyclophosph amide	yes; 6y	Alive	Coombs	(20)
CID-18	CID-G/AI	*RAG1*	a.M435V (1303AG) b.R699W (2095C¡T)	M	8y	AIHA; AN	skin	varicella infection; pneumonia; tuberculosis infections; parasitic infections	yes; pre-HCT	high-dose steroids; infliximab; and cyclophosph amide	yes; 8y	Died; 8y	Coombs; pANCA; ANA	(23)
SCID-1	SCID	*RAG1*	a. N766l b.K86VfsX33	M	13y	AIHA; AN	_	thyroiditis; hepatitis; urticaria	_	_	yes; 5m	Alive; 19y	Coombs	(5)
SCID-2	SCID	*RAG1*	a./b. K86VfsX33	F	1m	AIHA	_	_	yes; pre-HCT	_	yes;1y 3m	Alive 10y5m	Coombs	(5)
SCID-3	SCID	*RAG1*	del AA368–369	M	11m	AIHA; thrombocytopenia	_	hepatosplenomegaly; *CMV* infection	yes; pre-HCT	ganciclovir; foscarnet; and *CMV* hyperImmunoglobulin	yes;1y 4m	Alive	Coombs	(21)
SCID-4	SCID	*RAG1*	g.8366-8367insT, c.3198-3199insT, p.L1025FfsX39 g.6472C>T, c.1304C>T, p.R394 W	F	45d	AIHA;	_	hepatosplenomegaly; erythroderma; lymphadenopathy	_	_	_	_	Coombs	(22)
OS-1	OS	*RAG1*	g.8366-8367insT, c.3198-3199insT, p.L1025FfsX39	F	82d	AIHA; ITP;	_	_	_	_	_	_	Coombs	(22)
OS-2	Omenn syndrome	*RAG1*	g.8366-8367insT, c.3198-3199insT, p.L1025FfsX39 g.7481G>A, c.2313G>A, p.C730Y	M	60d	AIHA;	_	hepatosplenomegaly; erythroderma; lymphadenopathy	_	_	_	_	Coombs	(22)
OS-3	Omenn syndrome	*RAG1*	g.7862C>T, c.2694C>T, p.A857 V	F	120d	AIHA;	_	hepatosplenomegaly; erythroderma; lymphadenopathy	_	_	_	_	Coombs	(22)

AIHA (autoimmune hemolytic anemia), ITP (immune thrombocytopenia), AN (autoimmune neutropenia), *CMV* (cytomegalovirus), *RSV* (respiratory syncytial virus), TPO (thyroperoxidase antibody), HCT (Hematopoietic cell transplantation).

The clinical phenotype of *RAG* mutations manifested primarily as delayed combined immunodeficiency, characterized by granuloma or autoimmunity, known as CID-G/AI (n=18, 44%), followed by leaky/atypical severe combined immunodeficiency (SCID) (39%), and SCID (n=4, 10%), and Omenn syndrome (n=3, 7%). A breakdown of autoimmune complications revealed that 17 patients exhibited simple AIHA, eight had AIHA concomitant with immune thrombocytopenia (ITP), eight presented with AIHA and autoimmune neutropenia (AN), and six had AIHA in combination with AN and ITP. Eight children displayed granulomatous manifestations, seven of whom had cutaneous granulomas, and two presented with extracutaneous manifestations (affecting the lungs, liver, bone, pancreas, and testes in one case, and bone in another). Notably, one child manifested granulomatous changes exclusively in the lungs. Among other clinical symptoms, pneumonia (including a case of typical *Pneumocystis jirovecii* pneumonia in our patient) was reported in six patients, accompanied by various skin alterations (vitiligo, dermatitis, urticaria, chickenpox, alopecia, and scabies) in five patients, digestive disorders (enteritis, diarrhea, and ptosis) in four patients, urinary diseases (nephritis and nephropathy) in two patients, and neurological conditions (Guillain-Barré syndrome and demyelinating diseases) in four patients. Hepatosplenomegaly was identified in five patients. *CMV* (N=5), herpes virus (N=3), bacteria (N=3), and isolated cases of RSV, rotavirus, parasitic, tuberculosis, and fungi were also reported.

All 41 patients (100%) exhibited positive manifestations of Coombs’ autoantibody, along with other positive antibodies including anti-platelet antibodies (N=6), anti-neutrophil antibodies (N=5), anti-TPO antibodies (N=4), anti-IFN-α autoantibodies (N=3), and anti-nuclear antibodies (N=1).

Twenty-five patients were treated with IVIGs, 23 of these received treatment before hematopoietic stem cell transplantation (HSCT). Twelve patients were treated with rituximab, four of whom underwent plasma exchange. Twenty-eight patients who underwent HSCT had a median age at transplantation of 3.7 years (range, 6 months – 20 years). Of 11 patients that died, eight succumbed after HSCT and three before transplantation.

## 4 Discussion

In this report, we present a case of missense mutation in the *RAG1* gene causing AIHA as the first symptom, followed by *CMV* infection and severe *Pneumocystis jirovecii* pneumonia in a 1-year and 4-month-old female in China. The mutations were identified using a combination of whole-exome and Sanger sequencing. Additionally, we reviewed and summarized the clinical manifestations and genetic results of previously reported cases of AIHA associated with *RAG1* mutations.

RAG comprises a catalytic subunit, *RAG1*, and an essential cofactor, *RAG2*. The core of *RAG1* contains residues of the active site responsible for DNA cleavage and establishes extensive sequence-specific and non-specific interactions with RSS (Recombination Signal Sequence) and flanking DNA (24, 25). In combination with *RAG2, RAG1* forms a potent genome editing tool for lymphocytes by initiating V (D) J (V:Variable;D:Diversity;J:Joining) recombination (26). Polyclonal libraries of functional T and B lymphocytes expressing a diverse array of productive T cell receptor and B cell receptor rearrangement libraries were previously generated (26, 27). These libraries were strictly controlled to prevent dysregulation (28). However, in cases of dysregulation, ineffective mutations in *RAG1* and *RAG2* led to the SCID phenotype (29), as defined by the Primary Immune Deficiency Treatment Consortium in 2022 (30). Nevertheless, hypomorphic *RAG* mutations are associated with a range of clinical and immunophenotypes, including Omenn syndrome (24, 29, 30), leaky SCID and atypical SCID, which do not display typical Omenn syndrome characteristics, and SCID (31). Patients presented with a total T cell percentage of merely 0.5% (normal range: 55%–84%), and < 20% of CD4+ T cells were naive; these patients have been classified as exhibiting leaky/atypical SCID clinical phenotypes. The marked reduction in the total T cell percentage may be linked to impaired central T cell tolerance, subsequently facilitating the development of an anti-cytokine antibody restriction mode (30–33). The main clinical manifestations included AIHA and recurrent pneumonia, which were mainly caused by *CMV* and *Pneumocystis jirovecii*.

The ClinVar and dbSNP databases have documented over 300 mutation types in *RAG1*, including nonsense, frameshift, intra-frame deletions or insertions, and missense variants in *RAG1* and *RAG2*, which affect various protein domains. The two mutations we reported were located in exon 2 of *RAG1* [NM_000448.3(*RAG1*): c.2095C>T, p.(Arg699Trp)] and exon 2 of *RAG1* [NM_000448.3(*RAG1*): c.2690G>A, p.(Arg897Gln)]. While these mutations have been reported in gene banks, the clinical phenotype caused by these two mutations is still unclear. Regarding the first missense mutation, the patient’s mother was heterozygous and exhibited a phenotypic manifestation, whereas the father was wild-type. Whereas, the patient’s mother was wild type and phenotypically normal for the second genetic missense mutation, but the father was heterozygous, adhering to the genetic pattern of autosomal recessive inheritance, specifically compound heterozygous inheritance. According to the genetic test results, this condition was attributed to a rare missense mutation in *RAG1*. Following the guidelines established by the American College of Medical Genetics and Genomics and based on the clinical phenotype and familial analysis of patients, mutation 1 was deemed a pathogenic mutation, whereas mutation 2 was classified as a possible pathogenic mutation. However, substantial evidence exists for mutation 2, pointing towards its eventual classification as pathogenic, although it is currently insufficient to establish its pathogenicity definitively (34).

AIHA, a diverse condition marked by immune-mediated red blood cell destruction, presents with varying degrees of anemia (35). It is primarily classified based on antibody profiles and temperature reactions. IgG or IgG+C3d antibodies cause warm AIHA (wAIHA), while IgM results in cold agglutinin disease (CAD), causing approximately 20-25% of cases (30). The mixed form combines features of both wAIHA and CAD. The Coombs test, or direct antiglobulin test (36), is the diagnostic gold standard. Our patient’s positive results of direct Coombs, anti-C3D, and anti-IgG antibodies confirm the mixed AIHA diagnosis. However, if classified according to the cause, it can be classified into primary and secondary categories (37). Primary AIHA is often associated with immunodeficiency. The distinction between primary and secondary AIHA based on the presence or absence of an underlying disease or condition that exacerbates immune dysregulation and can be further elucidated through genetic sequencing (38). *RAG1* deficiency is a prototypical example of PID (Primary Immunodeficiency Diseases) with a wide range of phenotypic manifestations (38).

AIHA, caused by *RAG1* gene mutations, typically presents as severe, recurrent, and refractory anemia (30). However, the mechanism underlying AIHA may be related to the impairment of receptor editing due to *RAG1* gene mutations. The impairment of the receptor editing caused autoreactive immature B cells to exit the bone marrow, becoming transitional B (TrB) cells that express low levels of BAFF receptors (39), leading to elevated serum BAFF levels. In a lymphopenic environment, this further results in homeostatic proliferation of naive B cells (40). Qing Min and colleagues propose that this homeostatic proliferation leads to the generation of double-negative and memory B cells (41). The double-negative and memory B cells can efficiently differentiate into plasma cells and secrete large amounts of antibodies, including autoantibodies (41). However, it is currently unclear whether this finding is related to the development of AIHA, although elevated autoantibody levels have been observed in mouse models with *RAG* deficiency (26, 40). Furthermore, research has revealed that individuals harboring submorphological *RAG* mutations can generate a diverse array of autoantibodies after exposure to environmental stimuli (26, 30), which may alter the phenotypic expression of autoimmune symptoms. Among the 35 patients reviewed in the literature, except for Coombs-positive cases caused by AIHA, the autoantibodies in 11 patients were unknown, whereas anti-platelet antibodies, anti-neutrophil antibodies, anti-TPO antibodies, anti-IFN-α autoantibodies, and anti-nuclear antibodies were generated in other patients.

According to the World Health Organization, BCG vaccine is absolutely contraindicated in patients with SCID. However, since it is usually administered at birth, most SCID patients in countries where BCG is administered would have been vaccinated before their diagnosis of an immune deficiency (45). It is reported that one in every two SCID patients who receive the BCG vaccine will exhibit manifestations related to the vaccine. These manifestations, including disseminated complications, occur approximately 33,000 times more frequently than that in the general population, while local complications are about 400 times more common (42). In cases of SCID, where prophylactic treatments such as immunoglobulin replacement therapy and antimicrobial agents are commonly used, it is entirely appropriate to commence anti-mycobacterial treatment as early as possible following the diagnosis of SCID. The primary intervention affecting the survival of patients with SCID who had received the BCG vaccine, appears to be immune reconstitution through hematopoietic stem cell transplantation (HSCT). This may be related to the fact that HSCT itself is sufficient as an anti-BCG treatment (42). This implies that there had been neither a previous history of severe infections before age 1 year and 4 months in the patient, nor clinical manifestations of BCG disease or evidence of tuberculosis infection. During the hospital stay, the patient underwent two sputum T-NGS tests, which also did not reveal any signs of tuberculosis infection. Therefore, no clinical treatment was administered for BCG disease. However, the patient received IVIG as immunosupportive therapy, and antibiotic treatment.

In patients with *RAG* deficiency, HSCT, followed by genetic reconstitution, is the only definitive therapeutic option (27, 43). However, suitable donors for this purpose are not always available. According to our literature-based statistics, 28 patients underwent HSCT, with eight fatalities occurring post-transplantation and three before HSCT. Nonetheless, the literature suggests that only 50% of patients undergoing HSCT survive this procedure (44). Additionally, HSCT for CID administered before 3.7 months of age has been shown to yield more favorable outcomes (28, 38). However, the median age in our cohort was 3.75 years (range, 1 months – 20 years). This delay may stem from the need to arrange for patient transfers to other hospitals for treatment and the heightened risk of infections and tissue damage. It is well-established that infections and tissue damage can negatively affect HSCT outcomes (43). Thus, the management of patients with subformal RAG deficiencies is not as straightforward as that of patients with typical SCID. Autoimmune complications frequently necessitate immunosuppressive and immunomodulatory medications, further increasing the risk of infection. Accordingly, we observed a lack of response to first-(primarily IVIG and steroids) or second-line therapies (primarily rituximab) in most patients. Our patient was also treated with these drugs along with hormone and plasma exchange therapy. Although the hemolytic symptoms of the patient improved after treatment, serious opportunistic infections, giant cells, and *Pneumocystis jirovecii* were present during this period. The patient is now in good condition and is still undergoing relevant immunization counseling. Rituximab is a highly effective and safe alternative treatment for ITP and AIHA in patients with common variable immunodeficiency (41).When patients are diagnosed at a later age, there is an increased risk of developing graft-versus-host diseases. Furthermore, partial development of T and B cells requires a myeloablative preconditioning regimen to prevent graft rejection (46).

In conclusion, we report a case of *RAG1* mutation reported in China, which initially manifested as AIHA. We also present literature review that outlines the diverse clinical phenotypes observed in *RAG1* mutations presenting with AIHA. For such children, hormones and IVIG can be used as emergency treatments for AIHA; however, these drug control measures increase the risk of opportunistic infections, making HSCT the ultimate necessary choice.

## Data Availability

The original contributions presented in the study are included in the article/**Supplementary Material**. Further inquiries can be directed to the corresponding authors.

## Supplementary material

Supplementary Figure 1 Evidence of AIHA **(A)** Bone marrow cytology: Significant proliferation and activation of bone marrow, with decreased granulopoiesis, robust erythropoiesis dominated by mid-to-late stage erythroblasts, frequently exhibiting abnormal nuclei. These abnormalities include poikilocytosis (irregularly shaped red blood cell precursors), flower-like erythroblasts, polychromasia, and direct and indirect mitotic figures. **(B)** Value of hemoglobin levels, reticulocyte count, and reticulocyte percentage. Dotted lines indicate the reference range.

Supplementary Figure 2 Specific trends and values of inflammatory markers **(A)** C-reactive protein, interleukin-6 and procalcitonin. **(B)** White blood cell count and serum ferritin. Dotted lines indicate the reference range.
