# Supplementary figures and images for: Case report: Identification of a Chinese patient with RAG1 mutations initially presenting as autoimmune hemolytic anemia
Source: Front Immunol. 2024 Dec 10;15:1498066. doi: 10.3389/fimmu.2024.1498066 (PMC11666426; doi:10.3389/fimmu.2024.1498066)

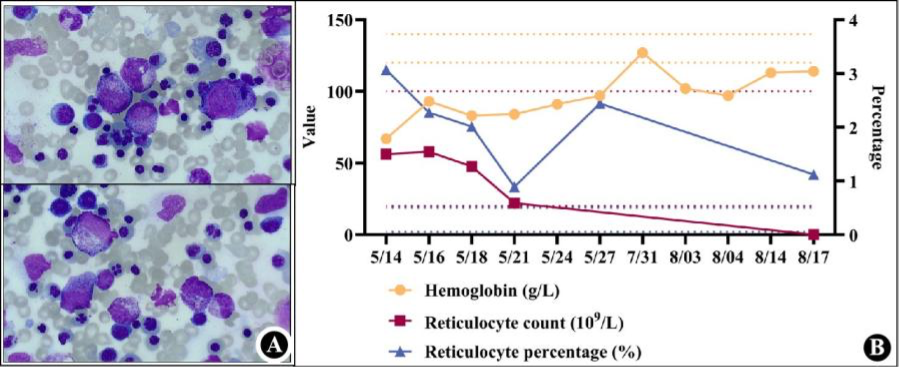

Supplement: Supplementary Figure 1 — Evidence of AIHA (A) Bone marrow cytology: Significant proliferation and activation of bone marrow, with decreased granulopoiesis, robust erythropoiesis dominated by mid-to-late stage erythroblasts, frequently exhibiting abnormal nuclei. These abnormalities include poikilocytosis (irregularly shaped red blood cell precursors), flower-like erythroblasts, polychromasia, and direct and indirect mitotic figures. (B) Value of hemoglobin levels, reticulocyte count, and reticulocyte percentage. Dotted lines indicate the reference range. [file Image1.tiff]

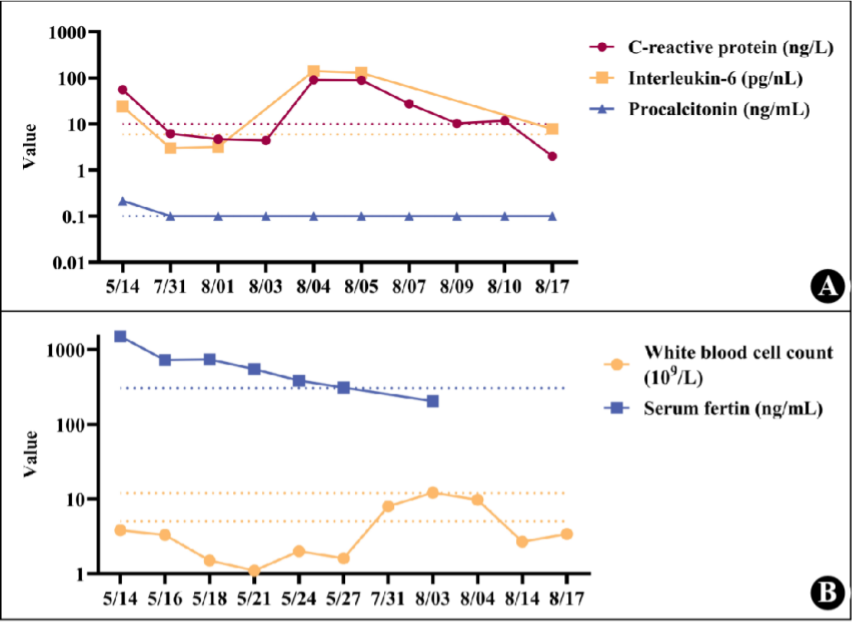

Supplement: Supplementary Figure 2 — Specific trends and values of inflammatory markers (A) C-reactive protein, interleukin-6 and procalcitonin. (B) White blood cell count and serum ferritin. Dotted lines indicate the reference range. [file Image2.tiff]
